# The regulation of competition and procurement in the National Health Service 2015–2018: enduring hierarchical control and the limits of juridification

**DOI:** 10.1017/S1744133119000240

**Published:** 2020-07

**Authors:** Dorota Osipovič, Pauline Allen, Marie Sanderson, Valerie Moran, Kath Checkland

**Affiliations:** 1 Department of Health Services Research and Policy, LSHTM, London, UK; 2 Luxembourg Institute of Socio-Economic Research, Esch-sur-Alzette, Luxembourg; 3 Division of Population Health, Health Services Research and Primary Care, University of Manchester, Manchester, UK

**Keywords:** Competition, health care, hierarchy, juridification, market, regulation

## Abstract

Since 1990, market mechanisms have occurred in the predominantly hierarchical National Health Service (NHS). The Health and Social Care Act 2012 led to concerns that market principles had been irrevocably embedded in the NHS and that the regulators would acquire unwarranted power compared with politicians (known as ‘juridification’). To assess this concern, we analysed regulatory activity in the period from 2015 to 2018. We explored how economic regulation of the NHS had changed in light of the policy turn back to hierarchy in 2014 and the changes in the legislative framework under Public Contracts Regulations 2015. We found the continuing dominance of hierarchical modes of control was reflected in the relative dominance and behaviour of the sector economic regulator. But there had also been a limited degree of juridification involving the courts. Generally, the regulatory decisions were consistent with the 2014 policy shift away from market principles and with the enduring role of hierarchy in the NHS, but the existing legislative regime did allow the incursion of pro market regulatory decision making, and instances of such decisions were identified.

## 1. Introduction

The Health and Social Care Act 2012 (HSCA 2012) codified the mechanisms governing provider competition in English National Health Service (NHS), leading to concern about the potential for the regulation of competition to become excessively juridified (Davies, 2013; Benbow, 2018). At its core the process of juridification involves a shift of power away from the executive branch of the government towards the judiciary. Thus, juridification impacts the relationship between law and politics in the liberal democracies (Aasen *et al*., 2014).

Juridification is a multidimensional phenomenon reflecting such aspects as an imposition of explicit and specific legal regulation in areas previously subject to executive discretion, as well as increased legal framing and conflict resolution with reference to law (Blichner and Molander, 2008). In the case of the governance of the internal market in the English NHS, juridification entails the introduction of legislative rules into a system traditionally governed by the principles of hierarchical ‘command and control’ and subject to political priorities. Such excess juridification with regards to the regulation of competition in the NHS was seen as unwelcome due to its potential to decrease accountability of commissioners and to increase private sector penetration of the internal NHS market, while strengthening market mechanisms (Davies, 2013).

In our previous analysis of regulatory activity conducted early in the HSCA 2012 regime we found limited evidence for juridification (Sanderson *et al.*, 2017). We argued that although the regulatory structures put in place by the HSCA 2012 had potential to strengthen the enforcement of competition in the NHS, in practice the extent of juridification was limited due to the development of systems within the NHS to manage and resolve issues internally where possible (Sanderson *et al.*, 2017). Following a rejection of the NHS hospital merger in Dorset made by the national competition regulator in 2013, the internal NHS regulator Monitor has adopted a role of a mediator between the NHS and the national competition authorities in relation to mergers to allow NHS mergers to progress. At the same time the tradition of settling procurement disputes without formal investigations or legal challenges continued (Sanderson *et al.*, 2017).

The HSCA 2012 represented a culmination of the expansion of the market principles into the NHS, with competition becoming not just a matter of internal policy administered through the NHS hierarchy, but that of law. Yet since the publication of the *Five Year Forward View* strategic plan (NHSE, 2014), the principle of cooperation has been elevated to become the preferred mechanism governing the supply of health care services. To date there have been no corresponding changes in the law to reflect this renewed policy emphasis on cooperation and integration, creating dissonance between the legal framework and commissioning policy and practice. Concurrently, there have been some developments in the regulatory framework in the form of extension of the Public Contracts Regulations 2015 to include health care services pulling in the opposite direction and having potential to further strengthen the market mechanisms within the NHS. As a result, there have been growing concerns among commissioners and policy makers about the law being out of step with the policy priorities and calls for changes to the legislation, culminating in the NHS Long Term Plan's list of required legislative changes (NHSE, 2019).

Given the recent policy turn to cooperation coupled with significant changes in the legal framework governing competition and procurement within the NHS there was a need to assess empirically recent regulatory decisions with regards to the enforcement of competition and procurement rules in the NHS to see if juridification has in fact been increasing, and whether legislative change is in fact necessary. As it currently stands, there are three public bodies with a mandate to make the regulatory decisions with regards to competition and procurement in the NHS: the sector economic regulator Monitor, now part of a body called NHS Improvement (Monitor/NHSI); the national competition regulator, the Competition and Markets Authority (CMA) and the courts. Examination of the actions of the regulators is important because they impact the actions of other actors within the system; the rules on the use of competition in the NHS are ambiguous, providers and commissioners look to the regulators for guidance on how to interpret them (Osipovič *et al.*, 2016). The way the regulatory decisions are made and enforced, and by whom, also indicates the distribution of power between different public bodies within the state.

We investigated how the legislative framework and policy guidance featured in the published regulatory decisions regarding competition and procurement in the NHS between 2015 and 2018. Due to the general limitations of the markets in health services and specific conditions of the NHS as a publicly funded system, the notion of competition in the NHS is balanced against the need to achieve wider social goals and is subject to sector specific rules. In this paper we analysed the extent to which such sector specific view of competition tailored to the conditions of the NHS internal market prevailed in the regulatory decisions, as opposed to principles of the general law on competition.

We found that the bulk of regulatory activity with regards to provider mergers and procurement disputes was carried out by the internal sector regulator Monitor/NHSI and was subject to sector specific rules. Our findings point to a continuing dominance and resilience of the hierarchical modes of control within the English NHS (see e.g. Allen, 2013). NHS hierarchy has managed to successfully resist juridification of regulation on competition and continued to apply internal, sector specific principles and mechanisms to settle procurement disputes and assure transactions. As far as the regulation of mergers and acquisitions between NHS providers is concerned, we found that the national regulator of competition the CMA has been made to adopt the sector specific rules. One of the strategies of resistance deployed by Monitor/NHSI was to decrease the visibility and transparency of their decision making processes around enforcement of competition. This had the effect of moving competition issues out of sight and thus down the policy agenda. This behaviour could be interpreted by the actors in the system as permission and encouragement to utilise the existing flexibilities within the law to downplay the competition principle in their decision making.

However, simultaneously we also found one area where the NHS hierarchy was not successful in keeping the regulation of competition an internal matter for the NHS. Our analysis showed an increasing role for the courts in adjudicating procurement disputes compared with the previous period. An extension of the Public Contracts Regulations 2015 (stemming from an EU directive) to health care services procurement has made it easier for providers to mount legal challenges based on procedural breaches of procurement processes. Yet in our view the scale of this juridification remains limited in comparison with the volume of the regulatory activity by the sector regulator Monitor/NHSI.

The structure of the paper is as follows. To situate our findings, we recap the regulatory framework put in place by the HSCA 2012 and discuss the recent (post-2014) changes in the legal framework and the policy direction. We outline our approach to data collection and present the findings of the analysis of the regulatory decisions. In the final section we trace the policy implications of the findings and situate the findings in the economic and governance literature.

## 2. The regulation of NHS competition under the HSCA 2012

Since the introduction of a quasi-market in the English NHS at the beginning of the 1990s, service provision is managed by commissioning bodies which procure services on behalf of patients. Clinical Commissioning Groups (CCGs) are currently the local bodies responsible for commissioning the majority of services, overseen by NHS England (NHSE). NHSE also has some direct commissioning responsibility for low volume/high complexity services, and for primary care services. Competition (either ‘for the market’ or ‘in the market’) is one of the mechanisms used by commissioners to allocate resources to service providers. Competitive behaviour within the quasi-market is heavily regulated by the economic regulator Monitor/NHSI and hierarchical aspects of the NHS extend to the supply side (Allen, 2013) (for further details on the NHS system in England see Appendix 1).

The premise that competition would benefit the NHS by increasing efficiency, quality and responsiveness to patients has been a cornerstone of the English health policy since the beginning of the 1990s (Jones and Mays, 2009; DH, 2010). Yet it was necessary for the NHS to adopt the sector specific regulation of competition due to the special nature of the market in relation to health care. Markets in health care do not conform to the features of the perfect market because of the significant asymmetries of information between providers of care and patients (or their agents) limiting patient choice, the need to regulate the quality of providers limiting the freedom to supply services and considerable market entry and running costs of providing services which limit the number of potential suppliers (Allen, 2013). In addition, markets do not give any regards to the principle of fair and free access to care according to need which is fundamental in the English NHS. In light of these limitations, while the main objective of general competition law is the maximisation of consumer welfare, in the sector specific law the objective to uphold and protect competition is balanced against other objectives, such as the achievement of wider social goals. In particular, the principle of competition in the NHS is not deemed to have an intrinsic value but is checked and balanced against that of patients' interests, such as equal access to services.

The HSCA 2012 extended competition law to apply to the planning and provision of the NHS services by making explicit links between the sector-specific rules governing competition within the NHS and existing national competition legislation (Sanderson *et al.*, 2017). In particular, the sector economic regulator Monitor (which became part of NHS Improvement on 1 of April 2016) was assigned concurrent powers with the national competition regulator the CMA in preventing anti-competitive behaviour and abuse of dominant position (Sanderson *et al.*, 2017). Any transactions between providers such as mergers, acquisitions or joint ventures are subject to review by the sector and/or national competition regulators. If a transaction involves at least one NHS Foundation Trust (NHS FT), a type of NHS provider with a greater degree of autonomy established under the Health and Social Care Act 2003 (Allen *et al.*, 2012), the CMA applies change of control, turnover and share of supply tests to determine whether a particular case results in a ‘relevant merger situation’ (CMA, 2014: 18). Under Section 79 of the HSCA 2012 and Section 30(1)(a) of the Enterprise Act 2002, if a merger involves NHS FTs, Monitor/NHSI has a duty to provide advice to the CMA on the associated patient benefits. According to the CMA's guidance, NHSI's advice is not binding on the CMA, but is given significant weight due to NHSI's expertise as the sector regulator (CMA, 2014: 46).

Another key regulatory mechanism which Monitor acquired under the HSCA 2012 is provider licencing. All providers of NHS-funded services are obliged to obtain a licence and obey the licence conditions. One of these (Condition C2 – Competition oversight) prohibits the provider from engaging in anti-competitive conduct (such as collusion) where this is detrimental to patient interests (Monitor, 2014*a*). Under the HSCA 2012 Monitor/NHSI has powers to take action against suspected breaches of provider licence conditions.

The secondary legislation introduced by the Procurement, Patient Choice and Competition Regulations (No. 2) 2013 (PPCCR 2013) suggested that competitive tendering is to be the preferred method of procuring clinical services. However, the PPCCR 2013 are complex. Regulations 2 and 3 stipulate that commissioners must procure services from providers who are most capable of providing services which secure needs of patients, improve quality, efficiency and integration of services, and provide best value for money. Commissioners are required to act transparently and proportionately and treat different types of providers equally and in a non-discriminatory way. Regulation 5 stipulates that commissioners can award a contract to a particular provider without running a procurement process if ‘the services to which the contract relates are capable of being provided only by that provider’. This regulation, alongside a number of different factors that ought to be taken into account during commissioning decisions, give latitude to commissioners as to whether to procure services competitively. Regulation 4 requires that commissioners consider patients' rights to exercise a choice of a provider, the integration of services and providers being able to compete as means of improving the quality and efficiency of provision of services.

The PPCCR 2013 give powers to Monitor/NHSI to investigate any alleged breaches of the procurement processes by CCGs or NHSE following a provider complaint or on its own initiative. Monitor/NHSI can declare the relevant arrangements ineffective and has broad powers to give directions as to what should be done by the commissioners but the regulator cannot order that the commissioners hold a competitive tender process.

The rules governing competition in the NHS and the arrangements for their enforcement are complex. While all the rules have a status of law and their breaches may be challenged in the courts, some rules such as HSCA 2012 and PPCCR 2013 apply only to health care and are referred to in this paper as ‘sector specific’ rules. These rules take into account specific features of the health care system in England and endorse a quasi-market principle of competition in health care (see Appendix 2 for a summary of the legal framework).

## 3. Changes in the regulatory environment since the publication of the *Five Year Forward View*

In October 2014 NHS England published a policy document, the Five Year Forward View, which outlined a strategic policy vision for the NHS underpinned by the closer cooperation between different providers (NHSE, 2014). This has led to some changes to the legal rules and policy guidance governing organisational behaviour in the NHS.

### 3.1 Policy direction

In contrast to the legislative framework, policy developments led by NHS England promoted cooperative modes of coordination and downplayed the role of competition. The Five Year Forward View policy plan envisaged a number of collaborative service delivery models requiring breaking down of organisational boundaries between primary, community, acute, mental health and social care sectors and between different providers within each service sector (NHSE, 2014). Other reports, e.g. the Dalton Review, discussed potential organisational forms for promoting greater collaboration and consolidation in the acute sector (Dalton, 2014). The turn towards greater integration and collaboration was reinforced by the national planning guidance issued in late 2015 (NHSE *et al*., 2015). This document stated that the NHS should concentrate on local, place-based planning to be achieved by cooperation between local stakeholders. The plans were to be called ‘Sustainability and Transformation Plans’ later renamed ‘Sustainability and Transformation Partnerships’. Guidance emphasised that ‘planning by individual institutions will increasingly be supplemented with planning by place for local populations’ and that the NHS was too focused on ‘organisational separation and autonomy that doesn't make sense to staff or the patients and communities they serve’ (NHSE *et al*., 2015). The policy direction away from competition was reiterated in the further policy documents (NHSE, 2017) and public statements by senior NHS leaders, with Simon Stevens (CEO of NHSE) stating on several occasions that competition was not appropriate for NHS organisations, which needed to reduce their ‘institutional self-interest’ in the interest of the whole local health economy (e.g. Dunhill, 2016; Thomas and West, 2017). This has culminated in the explicit call to repeal pro-competitive regulations in the NHS Long Term Plan (NHSE, 2019).

### 3.2 Public Contracts Regulations 2015

Simultaneously, there was a significant development in the legal framework. The Public Contracts Regulations 2006 were replaced by the Public Contracts Regulations 2015 (PCR 2015) which came into force in April 2016. The PCR 2015 implemented the EU Public Sector Procurement Directive (2014/24/EU), which provided rules for the procurement of goods, services and works above certain financial thresholds by public authorities. In the 2006 Regulations health care services fell under a so-called ‘Part B’ provision which meant that commissioners were able to award a contract without advertising where there was no cross-border interest (DH, 2016). The PCR 2015 abolished this exemption and introduced certain other requirements on commissioners with respect to competitive procurement of health care services. In particular, the procurement of health services above a certain threshold^1^ fell under a so-called Light Touch Regime (LTR).

The LTR process allowed for certain flexibilities in the procurement process but also imposed mandatory constraints. Rather than following a standardised procurement routes, health care commissioners have considerable flexibility in designing their own procedure, for instance by deciding the contract award criteria, splitting contracts into lots or carrying out market engagement. However, they are required to: advertise procurement opportunities by publishing contract or Prior Information notices in the Official Journal of the European Union and Contracts Finder, an online database of contract opportunities with the UK government and its agencies; to make all documents available in advance of the procurement; to publish contract award notices; and to include a standstill period. The latter refers to a period between notification of a contract award decision to bidders and actual conclusion of the contract, allowing any bidders to challenge the decision. Moreover, the tailor made procurement procedures under the LTR ought to be relevant, reasonable and proportionate and should not breach the equal treatment and transparency principles. Thus in contrast to the previous period, the PCR 2015 imposed on NHS commissioners a requirement to openly advertise and follow a transparent procurement process where the contract value exceeds the relevant threshold.

### 3.3 The regulators

In light of growing discrepancies between the existing legal framework and policy makers' emphasis on providers and commissioners working collaboratively, the regulators have been required to perform a difficult balancing act. At the same time the financial problems of many provider trusts became increasingly apparent (Gainsbury, 2017). These structural conditions delineated the space for the regulators' actions.

Firstly, in April 2016 Monitor/NHSI and the CMA signed a Memorandum of Understanding (MoU) which clarified the issues with respect to the exercise of their concurrent powers in monitoring anti-competitive transactions and conduct, and investigating the market and merger reviews (CMA and NHSI, 2016). In the MoU the sector and national regulators committed to working together and having ‘regard to the distinctive characteristics of the sector’ (CMA and NHSI, 2016: 2). The CMA committed to cooperation with the sector regulator and acknowledged that Monitor/NHSI's role is limited ‘to preventing anti-competitive behaviour that is detrimental to patients’ interests’ (CMA and NHSI, 2016: 8).

Secondly, Monitor/NHSI and NHSE strengthened their provider and commissioner monitoring systems. Monitor/NHSI exercised statutory powers of supporting, reviewing and approving transactions involving NHS trusts, including mergers and acquisitions, through an internal NHS transaction review process (NHSI, 2017). The aim of this process was to carry out an assessment as to whether a proposed transaction leads to improved performance such as releasing economies of scale or improving patient care (NHSI, 2017: 14). In cases involving merger and acquisitions, Monitor/NHSI also supports trusts in developing a sound rationale for the merger and helps to determine whether the CMA ought to be notified of the transaction.^2^

The transaction review process was aligned with the Integrated Support and Assurance Process (ISAP) developed jointly by the Monitor/NHSI and NHSE and designed to risk assess large and complex contractual arrangements that commissioners intend to put in place (NHSE and NHSI, 2017). The ISAP was introduced in 2016 to prevent a repeat of the collapse of the £726 m UnitingCare Partnership contract in Cambridgeshire and Peterborough (NAO, 2016; NHSE and NHSI, 2017; Waller and Carter, 2017). The contract awarded following a competitive tendering process to UnitingCare Partnership, a limited liability partnership between two local acute NHS FTs subcontracting with a range of other NHS and independent providers, was terminated after 8 months due to the failure to reach agreement on contract costs (NAO, 2016). The ISAP is focussed on mitigating financial risks and appraising whole system consequences of novel organisational arrangements in the NHS but was not designed to test for compliance with the procurement regulations (NHSE and NHSI, 2017).

It appears that following the policy turn to collaboration and the increasingly apparent financial problems of NHS trusts as a result of mounting deficits and cost-cutting pressures, the sector regulator Monitor/NHSI strengthened the scrutiny of the financial risks of proposed transactions and the optimisation of provision in the cash-strapped system. Concerns about ensuring that appropriate levels of competition exist within the system became of secondary importance to Monitor/NHSI.

Moreover, Monitor/NHSI was itself subject to restructuring. In 2018 Monitor/NHSI set up joint management structures and closer working with NHS England, the national executive body with commissioning oversight (Carding, 2018). Thus, the role of an independent, sector-based regulator of competition was being increasingly diluted and merged with the pre-existing NHS hierarchical means of control, perhaps reflecting the diminishing salience of market mechanisms in the NHS. This has culminated in explicit statements in the LTP that the two bodies should cease to act as arms length regulators (NHSE, 2019).

### 3.4 The courts

As the PCR 2015 extended the reach of the public contracts regulations to the procurement of health care services, this provided an avenue for increasing the role of the courts in adjudicating procurement disputes. NHS commissioners had to ensure that their procurement strategy complies with both the PPCCR 2013 and PCR 2015. In an event of non-compliance, providers could pursue a legal challenge based on the breaches of either regulations.^3^

In particular, under the regulations 95 and 96 of the PCR 2015 the remedies available to providers who launch a legal challenge include submitting a claim form which stops the public authority from going ahead with signing of the contract with a winning bidder. This results in an automatic suspension of the contract. The authority can apply to the court to have the suspension lifted. If the contract has already been signed, the court may find it ineffective based on procedural grounds such as failure to advertise as required or failure to abide by the standstill periods. The providers could also apply to the court for a judicial review of commissioners' actions on the basis of the PPCCR 2013. There was also a possibility of having a judicial review of the regulators' actions under the general principles of administrative law. Yet arguably the relative ease of mounting a legal challenge based on the procedural breaches under the PCR 2015 alongside a possibility to trigger an automatic suspension of the contract award process, made this an attractive option for providers to explore as a way of putting pressure on the commissioners.

## 4. Search strategy and data

We analysed the regulatory decisions made between August 2015 and October 2018 to provide continuity with our previous work covering the period from January 2009 to August 2015 (Sanderson *et al.*, 2017). Our approach was to focus on the regulatory decisions which were in the public domain as these were most likely to impact the practices and strategies of the actors in the system. Yet gathering empirical data on the regulatory activity with regards to the enforcement of the competition in the NHS has proved challenging. The first hurdle pertained to the fact that there was no single data source recording the outcomes of the regulatory decisions reached by the three regulatory bodies which have authority in this respect. Secondly, and more crucially, the regulatory bodies take different approaches to the level of transparency they afford to their regulatory decisions, with the sector regulator Monitor/NHSI being the least transparent compared with the CMA and the courts.

Given the ensuing complexity of collating relevant empirical data, our approach was to cast our net wide in terms of information available in the public domain. We reviewed Health Services Journal (HSJ), the main trade press title covering the health care sector in England, for relevant cases mentioning a potential or actual merger involving at least one NHS provider or a procurement dispute pertaining to provision of clinical services involving at least one public sector body (commissioner or provider). This allowed us to establish an initial database of cases covering the reviewed period. We proceeded by searching Monitor/NHSI's, CMA's, UK government's^4^ and the British and Irish Legal Information Institute's websites as well as utilising the Google search engine to refine the initial database, triangulate data sources and obtain follow-up information on relevant cases. We organised the data in an excel spreadsheet by case and kept updating it with new information relating to a particular case as and when it became available.

By following this method, we identified 29 cases of potential or approved mergers/acquisitions/joint ventures and 15 cases of commissioning/procurement disputes. These figures include all of the formal regulatory decisions reached by the CMA, Monitor/NHSI and the courts in the reviewed period which were publicly disclosed, i.e. details of the case were published on a particular authority's website, which are reported in Tables 1 and 2. The overall number of cases identified through our search strategy may underestimate the scale of the ongoing regulatory activity with regards to organisational transactions and procurement disputes. We expect that a number of additional cases pertaining either to procurement disputes or transaction reviews, particularly those which were dealt by Monitor/NHSI, have not been reported in the HSJ.

**Table 1.** Summary of regulatory decisions regarding competition in the NHS (August 2015–October 2018)

Case type	Decision-making body	Cases investigated	Outcome of the investigation and date
Acquisition	Monitor/NHS Improvement	Missing data	Missing data
	Competition and Markets Authority	University Hospitals Birmingham NHS FT and Heart of England NHS FT	Phase 1^a^ clearance 14 September 2017
		Derby Teaching Hospitals NHS FT and Burton Hospitals NHS FT	Phase 1 clearance 05 April 2018
Merger	Monitor/NHS Improvement	Missing data	Missing data
	Competition and Markets Authority	Ashford and St Peter's Hospitals NHS FT and Royal Surrey County Hospital NHS FT	Phase 2^a^ clearance 16 September 2015
		Central Manchester University Hospitals NHS FT and University Hospital of South Manchester NHS FT	Phase 2 clearance 03 August 2017
Joint Venture	Monitor/NHS Improvement	Missing data	Missing data
	Competition and Markets Authority	University Hospitals Birmingham NHS FT and HCA International Ltd	Approved without investigation 18 August 2017

a The CMA conducts a two-stage merger review process. It has discretion to conclude the review after initial Phase 1 or to instigate an in-depth Phase 2.

**Table 2.** Summary of regulatory decisions regarding procurement in the NHS (August 2015–October 2018)

Case type	Decision-making body	Cases investigated	Outcome of the investigation and date
Procurement	Monitor/NHS Improvement^a^	Northern Devon Healthcare NHS Trust/complex adult community services	NHSI found that CCG did not breach the PPCCR 2013 26 August 2015
		Care UK/NE London Treatment Centre	Investigation closed without reaching conclusion as to whether the rules were breached 05 August 2016
	Courts	*Injunctions*^b^:	
		Counted4 CIC vs Sunderland City Council	Council failed to lift Northumbria and Tyne and Wear NHS FT contract award suspension 18 December 2015
		Kent Community Health FT vs Dartford, Gravesham and Swanley CCG and Swale CCG	NHS trust failed to lift Virgin Care contract award suspension 27 May 2016
		Sysmex Ltd vs Imperial College Healthcare NHS Trust	NHS trust succeeded in lifting Abbott Laboratories Ltd contract award suspension 21 July 2017
		Lancashire Care FT & Blackpool Teaching Hospitals FT vs Lancashire County Council	Council failed to lift Virgin care contract award suspension 08 February 2018
		Central Surrey Health vs Surrey Downs CCG	CCG failed to lift IDEEA alliance (led by Epsom and St Helier University NHS Trust) contract award suspension 18 October 2018
		*Full Trials:*	
		Lancashire Care FT & Blackpool Teaching Hospitals FT vs Lancashire County Council	NHS trusts succeeded in challenging contract award to Virgin Care 22 June 2018
Commissioning		QSRC Ltd vs NHSE	Private provider did not succeed in challenging NHSE's commissioning decision 21 December 2015

a Cases refer to ‘Formal Investigations’ listed on Monitor/NHSI's website (https://www.gov.uk/government/collections/procurement-choice-and-competition-in-the-nhs-documents-and-guidance), accessed on 30 August 2018.

b Injunctions refer to procurement disputes under the PCR 2015.

## 5. Results

Despite a considerable volume of procurement disputes (15 cases) and merger cases (29 cases) identified by HSJ search, there were relatively few cases where the regulatory bodies made formal decisions which were made public (see Tables 1 and 2). The difference between the volume of transaction cases and disputes reported in the sector press and those cases which were brought to the attention of the regulators and resulted in a formal, publicly disclosed regulatory decisions implies that a substantial number of transactions and procurement disputes are being reviewed and resolved internally by Monitor/NHSI. The information on the precise number and details of cases handled by Monitor/NHSI is not publicly available.

We proceed by discussing the publicly disclosed regulatory decisions made by the relevant authorities with regards to mergers and acquisitions and procurement disputes and situate such decisions in a wider context of regulatory activity.

### 5.1 Mergers and acquisitions

Compared with the previous period (2009–2015) (see Sanderson *et al.*, 2017), we found that the national competition regulator has adapted its approach to the NHS. Following Monitor/NHSI's advice, the CMA acknowledged that in some circumstances greater consolidation of providers can bring substantial patient benefits. The CMA also acknowledged the limited financial budget of the NHS as a whole may dampen competitive pressures. All mergers between NHS FTs which were reviewed by the CMA were approved (see Table 1). We also found at least seven cases of completed mergers and acquisitions between the NHS trusts which were not reviewed by the CMA, despite involving NHS FTs. We can assume that these were approved by the sector regulator Monitor/NHSI. We also identified at least four cases where the proposed merger or acquisition between the NHS trusts was blocked by Monitor/NHSI.

Between August 2015 and October 2018 two merger and two acquisition cases were reviewed by the CMA (see Table 1). Where the CMA found that a merger or acquisition between two NHS FTs will result in the substantial lessening of competition (SLC), the relevant customer benefits were found to outweigh that (CMA, 2017*a*, 2018*a*). For instance, the CMA found that the proposed merger between Central Manchester University Hospitals NHS Foundation Trust and University Hospital of South Manchester NHS Foundation Trust would lead to SLC in the provision of some services (CMA, 2017*a*). It also found that prohibition of the merger would be the only effective remedy in this case. Nevertheless, the regulator concluded that such a prohibition would be disproportionate as it would result in the loss of substantial patient benefits which might arise as a result of the merger. In this case the CMA placed significant weight on NHSI's and local stakeholders' opinions which were strongly supportive of the merger. In the final report on the merger case, the CMA also acknowledged the systemic constraints that the NHS providers are facing (CMA, 2017*a*: 5).

The CMA's approach to handling the NHS mergers reflects a successful redefining of the role of competition in the NHS according to the sector specific rather than general understanding of the principle. Furthermore, our analysis of the cases of mergers reported in the HSJ implies that at least 11 cases of completed or proposed transactions between NHS providers have been handled by Monitor/NHSI without being referred to the CMA by using internal processes such as Monitor/NHSI's transaction review process (NHSI, 2017). Monitor/NHSI's transactions assurance processes were not open to public scrutiny, but press reports of the proposed mergers or acquisitions which were either blocked or allowed to proceed to completion by Monitor/NHSI suggest that these were judged based on the financial and clinical viability rather than any effects on competition.

Thus, the bulk of the regulatory activity with regards to mergers and acquisition in the NHS took place at the sectoral decision making level. The rules applied by the sector regulator in judging those decisions were not transparent but the outcomes suggest that financial and clinical viability concerns trumped any concerns about competition. This implies that both the sector and national regulators of competition utilised the flexibilities inherent in the legal framework on how the competition ought to be viewed.

### 5.2 Procurement and commissioning

In contrast to mergers and acquisitions, the application of sector specific understanding of competition has been less successful in handling procurement disputes. This was largely because of the rising salience of the courts, another actor external to the NHS, in the reviewed period. The new Public Contracts Regulations 2015 explicitly stated that these regulations, which govern procedural aspects of the procurement processes, now applied to the procurement of health care services. The PCR 2015 regulations have made it easier to challenge procurement decisions based on procedural grounds.

We outline the regulatory decisions and activity of Monitor/NHSI with regards to procurement disputes and discuss key court judgements.

#### 5.2.1 Monitor/NHSI investigations

Handling of procurement complaints by Monitor/NHSI under the PPCCR 2013 lacked transparency. In the reviewed period there were only two procurement disputes which have been escalated by Monitor/NHSI to a formal investigation (see Table 2). Both of them followed a provider complaint. Neither of them found any definitive breaches of the PPCCR 2013 (Monitor, 2015; NHSI, 2016; see also Sanderson *et al.*, 2017). For instance, in May 2016 Monitor/NHSI closed its investigation into the procurement of the North East London Treatment Centre without reaching a conclusion as to whether the procurement rules were breached (NHSI, 2016). An incumbent independent provider, Care UK, alleged that the CCGs breached the rules by offering the contract to the local NHS acute trust below national tariff price which allegedly posed risks to quality and safety of services. Monitor/NHSI accepted the undertakings by the CCGs to cease the procurement exercise and extend the contract for provision of services with Care UK followed by the re-procurement process (NHSI, 2016).^5^

There was no information publicly disclosed on Monitor/NHSI's website about formal investigations into procurement breaches conducted after 2016. The CMA (2016, 2017*b*, 2018*b*) concurrency reports did not mention any further Monitor/NHSI's regulatory activity. We also found no activity with respect to enforcement of the competition licence condition of the provider licencing regime. However, we found at least two procurement disputes where the involvement of Monitor/NHSI was explicitly mentioned by the HSJ reporters (Coggan, 2017; Thomas, 2017), and it seems likely that Monitor/NHSI was involved in more disputes due to its statutory role. However, the limited visibility of Monitor/NHSI's role in handling such disputes suggests that during the reviewed period Monitor/NHSI sought to avoid putting procurement disputes prominently on the agenda to align with the de-prioritisation of competition in the policy discourse.

#### 5.2.2 Court judgements

At the same time providers were using the levers available in the legal framework to challenge procurement decisions directly in the courts, based predominantly on alleged breaches of the PCR 2015. In the reviewed period there were two court judgements regarding procurement and commissioning complaints and five injunction decisions (see Table 2). There was no clear pattern in the outcomes of decisions on procurement disputes delivered by the courts.

Following a legal challenge by the local acute NHS trusts in May 2018 the High Court ruled that Lancashire County Council had not carried out the procurement in accordance with the PCR 2015 and ordered that the two bids from Virgin Care and local NHS acute trusts must be re-evaluated [(2018) EWHC 1589 (TCC) High Court]. The breach of the rules concerned the insufficiency of the reasons given by the council for the scores it awarded to the tenderers. Following the re-evaluation of the bids, the contract has been awarded to the original winner Virgin Care (Dunhill, 2018); it is not clear at the time of writing (January 2019) whether the NHS acute trusts will dispute this outcome once again.

Another case was brought by an independent provider of specialist radiosurgery services under the PPCCR 2013 in the pre-procurement dispute against NHSE which ceased to commission its services [(2015) EWHC 3752 (Admin) High Court]. The judicial review examined what it means to be an ‘existing provider’ in light of Monitor's 2014 guidance on commissioning radiosurgery services and any guarantees that stem from it (Monitor, 2014*b*). Overall, the court found that the NHSE did not breach the PPCCR 2013.

In the reviewed period, the courts also issued five contract injunction rulings under the PCR 2015 (see Table 2). Although the injunction decisions did not test the procedural or substantive issues with respect to procurement processes, by submitting a claim form the aggrieved providers stalled the contract award process which gave them leverage over commissioners.

In addition to the cases which were heard in the courts, we identified at least six other legal challenges reported in the HSJ that were dropped and/or settled out of court. The details of settlement between the parties and any potential role that Monitor/NHSI may have played in settling the disputes were not disclosed. Press reports indicated that some of such cases involved awards of financial damages to private providers (Moore, 2017) or a review or an extension of the contract (Gammie, 2018; Lintern, 2018).

## 6. Discussion and conclusions

Our analysis of the regulatory decisions has shown that the sector regulator Monitor/NHSI has succeeded in ensuring that the NHS merger control regime was subject to sector specific rules governing competition. This was achieved by cultivating a working relationship with the national regulator, the CMA, rather than through legislative change. Arguably, the attempts to bring procurement oversight under internal NHS control have proved less successful. Even though one can assume that the bulk of regulatory activity pertaining to procurement disputes remained within the remit of the sector regulator, Monitor/NHSI and hidden from public view, the litigation opportunities contained in the formal regulations made the actions of actors within the system more difficult to command and control. Furthermore due the independence of the judiciary, it was by definition impossible to reach a MoU on the way competition in the NHS ought to be viewed by the courts. The courts thus remained firmly outside of the sphere of influence of the sector regulator and applied the law.

These findings brought a number of issues to the fore. Firstly, a lack of transparency of Monitor/NHSI's actions in the reviewed period could be interpreted as an attempt to keep competition issues off the policy agenda, given that competition disputes became problematic for the sector regulator since the policy turn to collaboration. The insights from the policy analysis literature demonstrate that it is a prerogative of the powerful to keep conflicts which are potentially detrimental to the authority's dominant position or prevailing discourse from surfacing to the level of public visibility (Lukes, 2005). The regulator sent a signal to the actors that competition enforcement has moved down its agenda, tacitly giving permission to follow a more collaborative path in commissioning. On the other hand, the loss of visibility of regulatory decision making may have added to the actors' confusion about the prevailing rules in the system. The observed decrease in transparency in the application of rules by Monitor/NHSI also had negative implications for accountability of the NHS governing structures to the public (Benbow, 2018; Horton and Lynch-Wood, 2018). Secondly, deploying an ‘out of sight out of mind’ strategy could not fully succeed due to the independent role of the courts and the legal levers available to providers. In that sense our findings concur with the insights from the regulation literature showing that the introduction of formal legislation into the system hitherto governed by the sector specific principles reduces the power of the sector specific regulator.

However, we argue that the extent of and potential for juridification in this area is limited by a number of factors. It is important to stress that, based on the volume of regulatory activity, the sector regulator Monitor/NHSI dominated two other regulators – the CMA and the courts. This domination was largely hidden from public view, arguably due to the need to keep competition issues down the policy agenda. Moreover, as the judgements based on the PCR 2015 showed, the courts' remit was limited to scrutinising procurement processes for procedural flaws only. This remit of the courts is reflected in the solely procedural remedies that can be applied; considering whether competition is a desirable mechanism of procuring clinical services was outside that remit. For instance, in one of the injunction cases, the judge stressed that it was ‘not for the Court to second-guess the CCG's decision to put the services out for procurement’ [(2016) EWHC 1393 (TCC)]. Finally, it appears that the substantive issue which the PPCCR 2013 raises of how to weigh the factors of patient needs, quality, efficiency, service integration and value for money alongside aspects of transparency, proportionality and equal treatment of providers and whether to issue a tender at all, have not been tested in courts. It is not clear why to date there has been no judicial review of commissioners' decisions based on the alleged mistaken prioritisation of some factors over others, failure to tender or an unwarranted use of competitive tendering. Such challenges would be more consequential for curbing the power of sector regulator Monitor/NHSI than the current procedural cases. Thus, although we concur with Davies (2013) that the observed increased role of the courts strengthened the role of market principles in the NHS, we also argue that the solely procedural remit of the courts was one of the factors limiting the extent of juridification of the regulation of competition in the NHS.

The legal framework extended the scope of providers' strategies by offering an opportunity to bypass the sector regulator Monitor/NHSI in procurement disputes, but it did not offer any substantive assurances in favour of a particular commissioning approach or one type of a provider. Thus, winning a court case based on the procedural breaches in the procurement process did not guarantee winning the procurement process itself, as the Lancashire case demonstrated. Conversely, a mere threat of a legal action or obtaining an injunction triggering an automatic suspension of the contract could be sufficient in achieving the complaining provider's aims on the ground (e.g. Moore, 2017; Gammie, 2018; Lintern, 2018). This raises questions about the prudence of pursuing the legal route of settling procurement complaints by the NHS providers, given the high costs of litigation.

There is no doubt that the legal framework inflated the transactions costs associated with operating the internal market in the NHS by imposing national competition oversight of the NHS mergers and promoting litigation on procedural grounds. The fact that Monitor/NHSI effectively pre-approved mergers that it put forward to the CMA and that the CMA largely accepted the sector specific understanding of the competition, made the CMA's involvement in the NHS merger reviews somewhat redundant. The litigation on the grounds of procedural fairness of procurement processes had substantial costs to the taxpayer regardless of the outcome. Apart from direct costs of litigation, there were also increased transaction costs of having to repeat the procurement process in the case of a successful challenge. However, the more fundamental question about the appropriateness of competition in the NHS remained unexamined. Meanwhile, commissioners continued to be exposed to the threat of litigation, in particular from the independent providers (NHS Support Federation, 2017) while the regulators showed no commitment to support the commissioners should their decisions be subject to a legal challenge (NHSE and NHSI, 2017: 16; Timmins, 2018).

Overall, our analysis paints a complex picture of regulatory forces at play. In spite of years of marketisation reforms, the hierarchical mode of command and control in the NHS has been remarkably resilient. This may be due to hierarchy's better capacity to control rising costs of delivering health care in the system or to problematic dynamics of competition itself. NHS provider budgets are increasingly stretched and the costs of providing health care are rising in the system as a whole (Gainsbury, 2017; Charlesworth and Johnson, 2018). Competition in such a cash-strapped system cannot function properly because of the lack of excess capacity needed for the suppliers to compete with each other and for the purchasers to have an effective choice of a supplier (Dawson, 1995). Competition in the NHS thus remains ‘selective’ and subject to ‘differential regulation’ (Dawson, 1995: 16) rather than driven by the economic efficiency. Furthermore, competition in the markets has a tendency over time to transform into a monopoly or an oligopoly due to the original winners enjoying the benefits of control over asset specific investments and other suppliers attempting to ward off the hazards of this ‘fundamental transformation’ by vertically integrating (Williamson, 1985).

In this regulatory environment dominated by the NHS hierarchy we found only a limited space for juridification of the regulatory decisions, and that this was driven by the forces outside of the NHS hierarchy's control, namely the transnational EU legislation transposed into the PCR 2015 applicable to all public sector bodies procuring goods and services.

Arguably, the limits of juridification are also delineated by the particular set up of the English legal system. The courts in England see their role *vis-à-vis* the executive as limited to upholding and advancing a particular model of procedural justice and on the whole eschew adjudication on the substantive issues affecting health care provision (Syrett, 2011). The judicial reviews thus focus on examining the fairness of the procedures by which certain decisions were made by the public bodies rather than merits of those decisions *per se*. Litigation is also deemed an option of the last resort. This perhaps explains why more fundamental issues around the rationale and appropriateness of the market in the NHS so far have not been tested in the courts.

Thus, the way competition is regulated in the NHS is an example of the hybrid mode of English public sector governance characterised by the centralised political control over the way internal markets are allowed to operate, as well as hierarchical funding and accountability flows (Allen *et al.*, 2011). There is a lot of political discretion in the system helped by the fact that the law remains ambiguous. The quasi-market in the NHS functions on the state hierarchy's terms and is allowed to ebb and flow depending on the prevailing policy preference. The competition mechanism supported by an option of direct litigation remains residual and subservient to the decision making by the political hierarchy.

Nevertheless, the misalignment between the legal rules and government policy trickling down the NHS hierarchy remains uncomfortable for some actors and detrimental to the development of some policies. It is being tackled by utilising the existing flexibilities in the legal framework, in particular ‘doing less of [tendering and procurement]’ (Timmins, 2018). Our analysis has shown that the commissioners are more likely to face legal action if they decide to tender a service than if they decide not to tender. Equally, the discretion can be exercised based on the PCR 2015 which allows the commissioners to be creative with the procurement procedure, scoring and contract award criteria and effectively ‘design out’ unwelcome bids. Our review has shown that some aspects of the current regulatory framework allow for more flexibility and discretion than others. In particular, the merger approval process seems to pose fewer issues in terms of following sector specific rules on competition than procurement disputes which are harder for the sector regulator to control.

Alongside reliance on the existing flexibilities, the need for the legislative reform is being increasingly recognised (Darzi *et al*., 2018; DHSC, 2018; NHSE, 2019). The NHS Long Term Plan published in January 2019 calls for a legislative change to enable more rapid system transformation towards collaborative commissioning and provision. In particular, the plan proposes to remove the CMA's oversight of the NHS mergers and Monitor/NHSI's competition enforcement roles (NHSE, 2019). It proposes among other things that commissioners should be free to decide the circumstances in which they should use procurement and that the NHS should be exempt from ‘wholesale inclusion in the Public Contract Regulations’ (NHSE, 2019: 114). Instead, the NHS policy makers propose to introduce sector specific statutory guidance for the NHS and to pursue closer integration between NHSE and Monitor/NHSI.

The proposed changes are consistent with the conclusions of our analysis of the consequences of the current regulatory framework. They signal continuing shift away from the market principles, blurring of the purchaser provider split and strengthening of the executive, sector specific powers of control over the system. They amount to acknowledging, at least in the policy terms, that the NHS is not a market and therefore should not be subject to or oblige to endorse the market principles. These are all welcome developments. However, it is important to note that to deliver on these proposals will require complex legislative processes involving many stakeholders with diverse interests and agendas. Agreeing the changes to the HSCA 2012 and passing them through parliament will be a challenging task, given the current political context (Timmins, 2018). The PCR 2015, due to its wider applicability to public sector and its transnational provenance, may be even more difficult to amend. The likelihood of amending the PCR 2015 will also depend on any trade agreements that the UK government would wish to pursue after leaving the European Union. Thus, it remains to be seen which of these legislative proposals and in what form materialise in law.

## Footnotes

1 The threshold is currently set at £615,278 (CCS, 2017).

2 The trusts decide whether or not to notify the CMA of a proposed transaction. If the trusts decide not to notify the CMA (even though their merger would be deemed reviewable by the CMA), the CMA may initiate review up to 4 months following completion of the transaction (NHSI, 2017: 12).

3 Under the Regulation 17 of the PPCCR (no. 2) 2013 procedural issues cannot be dealt jointly under the PCR 2015 and PPCCR (no. 2) 2013. If the provider brings action under the PCR 2015 they may not bring one about the same issue under the PPCCR (no. 2) 2013.

4 In particular, we searched the https://www.gov.uk/government/groups directory for evidence of completion of mergers and acquisitions identified through the HSJ search.

5 In September 2017 Care UK won the re-procurement process and was awarded a 3-year contract to run the services at the NELTC (Clover, 2017).

## Supplementary material

For supplementary material accompanying this paper visit https://doi.org/10.1017/S1744133119000240. click here to view supplementary material
