# The regulation of competition and procurement in the National Health Service 2015–2018: enduring hierarchical control and the limits of juridification – ADDENDUM

**DOI:** 10.1017/S1744133119000264

**Published:** 2020-07

**Authors:** Dorota Osipovič, Pauline Allen, Marie Sanderson, Valerie Moran, Kath Checkland

**Keywords:** Competition, health care, hierarchy, juridification, market, regulation, addendum

This article when published neglected to mention Dr Valerie Moran's second affiliation. She is affiliated with the Luxembourg Institute of Health, Strassen, Luxembourg and the Luxembourg Institute of Socio-Economic Research, Esch-sur-Alzette, Luxembourg.
