# Supplementary material for: The regulation of competition and procurement in the National Health Service 2015–2018: enduring hierarchical control and the limits of juridification
Source: Health Econ Policy Law. 2019 Sep 6;15(3):308–24. doi: 10.1017/S1744133119000240 (PMC7525100; doi:10.1017/S1744133119000240)
Supplement: Supplementary file 1 [file S1744133119000240sup.zip › S1744133119000240sup001.docx]

**APPENDIX 2.** Competition and procurement rules applicable to the NHS and enforcing bodies (August 2015 - October 2018)

| Rules | Status | Applicability | Enforcing body |
| --- | --- | --- | --- |
| Competition Act 1998 | Law | All aspects of the economy | Competition and Markets Authority (concurrent responsibilities with Monitor/NHS Improvement)// Courts |
| Enterprise Act 2002 | Law | All aspects of the economy | Competition and Markets Authority (concurrent responsibilities with Monitor/NHS Improvement)// Courts |
| Health and Social Care Act 2012 | Law | Commissioners and providers of NHS services in England | Monitor/NHS Improvement// Courts |
| Procurement, Patient Choice and Competition Regulations 2013 | Law | Commissioners and providers of NHS services in England | Monitor/NHS Improvement// Courts |
| Public Contracts Regulations 2015 | Law | All public authorities in England | Courts |
