# Supplementary material for: The regulation of competition and procurement in the National Health Service 2015–2018: enduring hierarchical control and the limits of juridification
Source: Health Econ Policy Law. 2019 Sep 6;15(3):308–24. doi: 10.1017/S1744133119000240 (PMC7525100; doi:10.1017/S1744133119000240)
Supplement: Supplementary file 1 [file S1744133119000240sup.zip › S1744133119000240sup002.docx]

**APPENDIX 1.** Relationships between commissioners, providers and regulators in the NHS in England as of December 2018


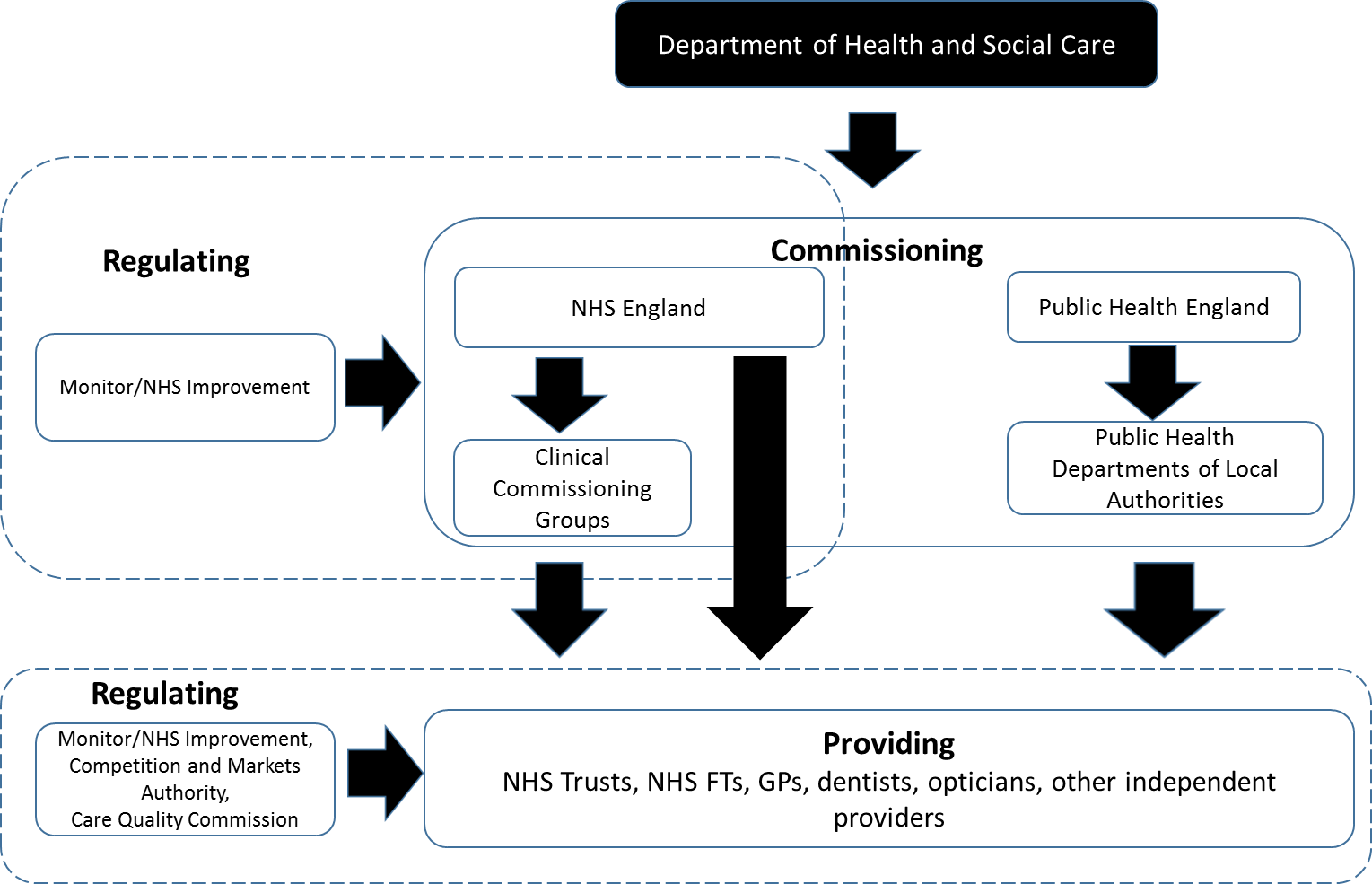


Since the introduction of a quasi-market in the English NHS at the beginning of the 1990s, service provision is managed by commissioning bodies which procure services on behalf of patients. Clinical Commissioning Groups (CCGs) are the local bodies responsible for commissioning the majority of services, overseen by NHS England (NHSE). NHSE also has some direct commissioning responsibility for low volume/high complexity services, and for primary care services (see Figure above). NHSE (formally named the NHS Commissioning Board in the HSCA 2012) is an Arm’s Length Body, operating under a mandate from the Department of Health and Social Care (Checkland et al., 2013).

The system is funded through general taxation and the services are free at the point of delivery for UK residents. Competition (either ‘for the market’ or ‘in the market’) is one of the mechanisms used by commissioners to allocate resources to service providers. Allocative decisions are based on the consideration of the rights and needs of the collective, as well as the individual. In order to encourage the diversification of providers, the entry of independent sector providers has been encouraged as well as the establishment of a new type of NHS provider organisation with a greater degree of autonomy under the Health and Social Care Act 2003– NHS Foundation Trusts (NHS FT) (Allen et al., 2012). The resultant system is referred to as a ‘quasi-market’ which aims to combine the supposed advantages of competition between suppliers with the safety of retaining public funding to protect fairness in access to care (Allen, 2013). Despite this diversification of providers and incentives for competition, evidence suggests that the hierarchical aspect of the NHS extends to the supply side (Allen, 2013).

Competitive behaviour within the quasi-market is heavily regulated, with competition on the basis of price limited. To enter the quasi-market of the NHS, providers need to be registered with the quality regulator, the Care Quality Commission and have a provider licence issued by the economic regulator Monitor/NHSI. Their clinical and financial performance is then continuously monitored by these regulators. Monitor/NHSI also has a duty to ensure that the commissioners of health care services – the CCGs and NHSE – use competition appropriately.
